# Pathological mechanisms of avian viral oncogenesis: The central role of MicroRNA

**DOI:** 10.1016/j.psj.2026.106869

**Published:** 2026-03-27

**Authors:** Ahmed Fotouh, Rania M. Elbatawy, Ibrahim Eldaghayes, Eman Abd El‑Menamm Shosha

**Affiliations:** a Pathology and Clinical Pathology Department, Faculty of Veterinary Medicine, New Valley University, Kharga, Egypt; b Pathology Department, Faculty of Veterinary Medicine, Benha University, Moshtohor, Toukh 13736, Qaluiobiya, Egypt; c Department of Microbiology and Parasitology, Faculty of Veterinary Medicine, University of Tripoli, Tripoli P.O. Box 13662, Libya; d Virology Department, Faculty of Veterinary Medicine, New Valley University, Kharga, Egypt

**Keywords:** Oncogenic, Virus, Pathology, microRNA, Pathogenesis

## Abstract

Avian oncogenic viruses represent a major cause of neoplastic and immunosuppressive diseases in poultry, leading to substantial economic losses worldwide and providing valuable models for studying virus-induced tumorigenesis. Among these viruses, Marek’s disease virus, avian leukosis virus, and reticuloendotheliosis virus induce lymphoid and hematopoietic malignancies through distinct yet convergent molecular mechanisms. Increasing evidence indicates that microRNAs and their viral homologs are central regulators linking viral infection to oncogenic transformation, immune dysregulation, and pathological outcomes. This review provides an integrated overview of microRNA's role in the pathogenesis and pathology of avian oncogenic viral diseases, emphasizing its contributions to lymphocyte proliferation, survival, immune modulation, and tumor development. We discuss the unique case of Marek’s disease virus, in which a viral microRNA ortholog is indispensable for tumor induction in vivo, representing the first direct demonstration of a virus-encoded microRNA acting as a primary oncogenic factor in a natural host. In contrast, avian leukosis virus and reticuloendotheliosis virus exploit host microRNA pathways through insertional mutagenesis and *NF-κB-*mediated transcriptional activation, respectively, contributing to tumor progression in a context-dependent manner. The review further correlates molecular findings with gross and microscopic pathological lesions. It highlights how microRNA–driven regulatory networks shape disease severity, tissue tropism, and tumor morphology. So, these insights establish microRNA as a conserved molecular hub in avian viral oncogenesis and underscore its relevance for understanding viral tumor pathology, improving differential diagnosis, and advancing comparative oncology across species

## Introduction

The global poultry industry represents one of the most significant segments of animal agriculture, supplying a major proportion of the world’s meat and contributing substantially to food security and rural livelihoods (Abd-El Hamed et al., 2025). However, this economically vital industry is continually challenged by infectious diseases, among which oncogenic viruses are particularly detrimental (Nair et al., 2020).

**Fig. 1:**
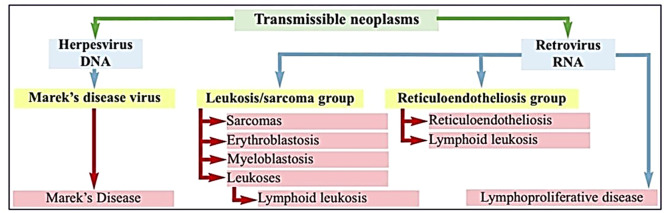
principal transmissible neoplasms of poultry (Brugère-Picoux et al., 2015).

Tumor-inducing viruses such as Marek’s disease virus (MDV), avian leukosis virus (ALV), and reticuloendotheliosis virus (REV), these transmissible neoplasms can infect chickens and cause a spectrum of neoplastic and immunosuppressive conditions that undermine both productivity and flock health (Fig. 1) (Witter et al., 2010). Marek’s disease alone has been associated with annual economic losses exceeding USD 1–2 billion worldwide, due to mortality, condemnations, reduced growth performance, and costs associated with vaccination and control measures (Khaled et al., 2024). In addition to direct production losses, oncogenic viral diseases can lead to immunosuppression, increased susceptibility to secondary infections, decreased feed conversion efficiency, reduced egg production, and substantial expenditures on prevention and mitigation strategies (Fotouh et al., 2020).

Most oncogenic viruses possess mechanisms that ensure their stable maintenance in host cells by equal segregation of viral genomes into daughter cells during cell division (Zavala et al., 2006). Virus-induced cell immortalization may occur through direct or indirect mechanisms. Direct mechanisms involve deregulation of cellular oncogenes or tumor suppressor genes, either through integration of the viral genome into the host genome or through the expression of viral oncogenes, such as those encoded by herpesviruses, which disrupt key regulators of cell cycle control and genomic stability, ultimately promoting DNA damage and cellular transformation (Chakraborty et al., 2019). Indirect mechanisms of oncogenesis include virus-induced chronic inflammation, immune-mediated tissue damage, and virus-associated immunosuppression, all of which impair antitumor immune surveillance and create a permissive environment for malignant progression (Xiang et al., 2022).

These oncogenic viruses have played a pivotal role in shaping our understanding of fundamental molecular mechanisms underlying cancer (Yue and Lopez, 2020). Landmark discoveries such as the identification of viral oncogenes, including *v-src* in Rous sarcoma virus and *v-myc* in avian retrovirus *MC-29*, were instrumental in establishing the concept of oncogenes and elucidating their roles in cellular transformation (Zhang et al., 2021). Further insights into tumor biology emerged from studies on multifunctional viral proteins, exemplified by SV40 large T antigen, which led to the discovery of key tumor suppressor proteins such as *p53* and retinoblastoma protein, thereby advancing knowledge of cell cycle regulation and neoplastic progression (Filipowicz et al., 2008).

At the molecular level, the progression and pathogenicity of these viral diseases involve complex interactions between viral genomes and host cellular processes. Among the regulatory mechanisms implicated in these interactions, microRNAs (miRNAs) are small endogenous non-coding RNA molecules, approximately 20–24 nucleotides in length, that play a fundamental role in post-transcriptional gene regulation (Volinia et al., 2010). Since their initial discovery, miRNAs have been identified across a wide range of organisms, including animals, plants, and viruses, and their biogenesis and regulatory mechanisms are now well established (Lin and Gregory, 2025). miRNAs are generated through a multistep maturation process that involves nuclear transcription, enzymatic processing, cytoplasmic export, and the incorporation of the guide strand into the RNA-induced silencing complex (RISC), enabling sequence-specific regulation of target mRNAs (Svoronos et al., 2016). Through mRNA degradation or translational repression, miRNAs regulate diverse biological processes, including cell proliferation, differentiation, apoptosis, immune responses, viral infection, and tumorigenesis (Hsin et al., 2018). In poultry, the oncogenic viruses disrupt immune function, enhance susceptibility to secondary infections, reduce vaccine efficacy, and cause substantial economic losses (Lu et al., 2015). Increasing evidence indicates that miRNAs are critically involved in the pathogenesis of these viral diseases by modulating viral replication, host immunity, and tumor development, highlighting their importance as key regulators in avian viral oncogenesis (Vojtechova, and Tachezy, 2018). In this review, we highlight the significant roles of miRNAs and their virus-encoded orthologs in neoplastic transformation by avian oncogenic viruses.

## MicroRNA

The history of miRNA as a multifunctional regulatory molecule is deeply rooted in early studies of virus-induced lymphomagenesis in poultry, particularly investigations of lymphoid tumors arising in chickens infected with highly oncogenic avian leukosis virus (Seddiki et al., 2014). Initial work on ALV-induced lymphomas identified retroviral insertional activation of classical proto-oncogenes such as *c-myc* and *c-myb* as primary drivers of neoplastic transformation; however, subsequent analyses revealed an additional recurrent integration site, designated *bic* (B-cell integration cluster), which was strongly associated with oncogene activation, tumor progression, and metastasis (Elmeligy et al., 2024). At the time of its discovery, the biological significance of bic was unclear, as microRNAs had not yet been identified; nonetheless, the observation that bic encoded a non-protein-coding RNA marked an important conceptual shift (Gottwein et al., 2007). This locus was later shown to generate the precursor hairpin structure from which miRNA is processed, establishing *bic* as the genomic origin of one of the most intensively studied oncogenic microRNAs (Sin et al., 2013). Following this discovery, extensive experimental and clinical studies across multiple species confirmed a strong association between aberrant miRNA expression and a wide range of lymphoid malignancies, firmly positioning miRNA as a key oncomiRNA (Gottwein et al., 2011). Functional evidence supporting this role emerged from transgenic mouse models engineered to overexpress miRNA, which consistently developed aggressive B-cell lymphomas accompanied by downregulation of critical tumor-suppressive target proteins (Burnside and Morgan, 2011), including SHIP1 and CEBPβ, thereby demonstrating a direct causal relationship between miRNA dysregulation and malignant transformation (Yao and Nair, 2014). Beyond its oncogenic capacity, miRNA plays an essential role in normal immune system function, acting as a central regulator of immune activation, lineage commitment, and cellular differentiation (Skalsky et al., 2007). It is critically involved in both innate and adaptive immune responses, modulating signaling pathways via inducible targets such as SOCS1, thereby fine-tuning cytokine signaling and inflammatory responses (Costinean et al., 2006). In B lymphocytes, miRNA is required for effective antigen-driven responses and plasma cell differentiation, in part by regulating the transcription factor PU.1, which governs gene expression programs during B-cell maturation and influences activation-induced cytidine deaminase (AID)–mediated processes essential for antibody diversification (Vigorito et al., 2007). Despite these indispensable physiological roles, miRNAs are predominantly recognized for their pathogenic potential, as sustained or deregulated expression disrupts immune homeostasis, promotes unchecked lymphocyte proliferation, inhibits apoptosis, and creates a cellular environment conducive to oncogenic transformation (Pekarsky and Croce, 2010; Fotouh et al., 2025).

Although the core mechanisms of miRNA biogenesis are broadly conserved among eukaryotes, several studies have characterized the molecular components and regulatory features of the miRNA maturation pathway specifically in chickens (Michlewski and Cáceres, 2019). In avian cells, miRNA genes are primarily transcribed by RNA polymerase II, producing long primary transcripts (pri-miRNAs) that contain characteristic stem–loop structures similar to those described in mammals. These pri-miRNAs undergo nuclear processing by the microprocessor complex, composed mainly of the RNase III enzyme Drosha and its essential cofactor DGCR8 (DiGeorge syndrome critical region 8), which recognizes the hairpin structure and cleaves the pri-miRNA to generate precursor miRNAs (pre-miRNAs) approximately 60–70 nucleotides in length (Wu et al., 2019). Studies in chicken embryonic fibroblasts and avian immune cells have confirmed that both Drosha and DGCR8 homologs are expressed and functionally conserved in avian species, indicating that the initial steps of miRNA maturation closely parallel those described in mammals (Burnside and Morgan, 2011; Hicks and Liu, 2013).

Following nuclear processing, the pre-miRNA is transported from the nucleus to the cytoplasm through the Exportin-5/Ran-GTP–dependent transport system, which has also been identified and functionally characterized in chickens. Exportin-5 recognizes the two-nucleotide 3′ overhang generated by Drosha cleavage and facilitates the efficient nuclear export of the pre-miRNA hairpin (Triber et al., 2019). In the cytoplasm, the RNase III enzyme Dicer, together with accessory proteins such as TRBP (TAR RNA-binding protein) or related double-stranded RNA-binding proteins, cleaves the pre-miRNA into a short double-stranded miRNA duplex of approximately 21–24 nucleotides (Michlewski and Cáceres, 2019). One strand of this duplex, designated the guide strand, is subsequently incorporated into the RNA-induced silencing complex (RISC), whose catalytic core is formed by Argonaute proteins (AGO1–AGO4). Functional studies in chicken immune and lymphoid cells have demonstrated that Argonaute-dependent RISC complexes mediate post-transcriptional repression through mRNA degradation or translational inhibition in a manner comparable to mammalian systems (Chen et al., 2020).

Avian miRNA biogenesis exhibits several distinctive regulatory features. For example, genome-wide analyses in chickens have identified numerous chicken-specific miRNAs and lineage-restricted miRNA clusters that are absent in mammals, suggesting species-specific expansion and functional specialization of the avian miRNA repertoire (Shosha et al., 2026). Additionally, viral infections in poultry, particularly those caused by oncogenic viruses, can modulate the expression of host miRNA biogenesis components, including Dicer and Argonaute proteins, thereby altering the global miRNA landscape in infected cells (Yao and Nair, 2014; Zhou and Zheng, 2019). These virus-induced alterations in the avian miRNA processing machinery may contribute to immune dysregulation, enhanced viral persistence, and oncogenic transformation.

## MicroRNA and oncogenic viruses

Avian oncogenic viruses have emerged as a powerful biological model for elucidating the roles of miRNAs and their viral homologs in virus-induced lymphoid transformation, immune dysregulation, and tumor progression, revealing a deeply conserved oncogenic regulatory axis that operates across distinct viral families and host species (Gimeno et al., 2008).

Among these viruses, Marek’s disease virus type 1 (MDV-1) represents one of the most extensively studied examples of viral oncogenesis in poultry, causing Marek’s disease, a highly prevalent lymphoproliferative disorder characterized by rapid-onset T-cell lymphomas affecting multiple visceral organs (Faiz et al., 2017), infiltration of peripheral nerves leading to paralysis, and profound immunosuppression, resulting in estimated annual economic losses approaching US$ 2 billion worldwide (Khaled et al., 2024). MDV-1 is an oncogenic alphaherpesvirus of the genus Mardivirus, which also includes the non-pathogenic Gallid herpesvirus 3 and the antigenically related herpesvirus of turkey (HVT), both of which are exploited as vaccines and viral vectors (Nair et al., 2020). Despite nearly five decades of intensive vaccination using strains such as CVI988 (Rispens), MDV-1 continues to evolve toward increased virulence, giving rise to virulent (vMDV), very virulent (vvMDV), and very virulent plus (vv+MDV) pathotypes, indicating that viral regulatory mechanisms beyond classical protein-coding oncogenes play a critical role in disease persistence and progression (Witter et al., 2010). Representative strains include Md5 and GA for vMDV, RB-1B and 648A for vvMDV, and strains such as 686 and 584A for vv+ MDV, each demonstrating progressively greater tumor incidence, earlier disease onset, and increased mortality (Zhou and Zheng, 2019). Importantly, increasing virulence among these pathotypes has been associated with significant alterations in the expression profile of viral microRNAs, particularly those located in miRNA cluster-1 of the MDV-1 genome (Xu et al., 2011).

Comparative transcriptomic analyses have demonstrated that highly virulent strains such as RB-1B (vvMDV) exhibit markedly elevated expression of several cluster-1 miRNAs, including MDV-*miR-M4, miR-M2, miR-M3,* and *miR-M12*, compared with less virulent strains or vaccine viruses (Morgan et al., 2008; Hicks and Liu, 2013). Among these, MDV-*miR-M4* is consistently the most abundant viral miRNA in tumor tissues and MD-transformed cell lines, frequently accounting for the majority of total viral miRNA reads (Yao et al., 2008). Functional studies have further demonstrated that deletion of cluster-1 miRNAs from the genome of the vvMDV strain RB-1B abolishes tumor formation in experimentally infected chickens, providing direct evidence that these miRNAs contribute significantly to the oncogenic phenotype of highly virulent MDV strains. These findings strongly support the concept that miRNA cluster-1 acts as a virulence-associated regulatory module, enhancing the transforming capacity of MDV-1 in highly pathogenic pathotypes (Mays et al., 2019).

A breakthrough in understanding MDV-1 oncogenicity came with the discovery that the virus encodes an extensive repertoire of miRNAs, first identified in chicken embryo fibroblasts infected with the highly virulent RB-1B strain and subsequently confirmed in MSB-1 lymphoblastoid cell lines derived from MD lymphomas (Gimeno et al., 2014). These studies revealed at least 14 miRNA precursors that generate 26 mature miRNAs, clustered within three distinct genomic loci located in the inverted repeat regions of the MDV-1 genome (Faiz et al., 2016). While these miRNAs exhibit a high degree of sequence conservation across MDV-1 strains, their expression levels vary markedly among strains of differing virulence, particularly those encoded within cluster 1, suggesting a direct association between miRNA expression patterns and oncogenic potential (Mays et al., 2019).

Functional studies using reverse genetics provided definitive evidence for this hypothesis, demonstrating that deletion of the cluster 1 miRNAs from the RB-1B genome completely abolishes viral oncogenicity in vivo (Zhou and Zheng, 2019). Within this cluster, MDV-*miR-M4* was identified as the dominant oncogenic determinant, accounting for more than two-thirds of the total miRNA reads detected in tumor cells. MDV-*miR-M4* was subsequently shown to be a functional ortholog of cellular miRNA, sharing an identical seed sequence and the capacity to regulate many of the same target genes involved in lymphocyte survival, proliferation, apoptosis, and immune signaling (Ibrahim et al., 2025).

The expression of MDV-*miR-M4* and other cluster 1 and 2 miRNAs is driven by a single epigenetically active promoter characterized by activating histone modifications and hypomethylation, ensuring sustained miRNA expression during infection and transformation (Bondada et al., 2019). Comparative analyses of miRNA orthologs, including human miRNA, *KSHV-encoded miR-K12-11,* and *MDV-miR-M4*, revealed a conserved set of shared targets in both avian and human transformed cells, including regulators such as *JARID-2* and *NF-κB*-inducing kinase, underscoring the evolutionary conservation of miRNA–mediated oncogenic pathways across approximately 300 million years of divergence between birds and mammals (Zhou and Zheng, 2019).

The critical role of MDV-*miR-M4* in lymphomagenesis was unequivocally confirmed through in vivo experiments showing that deletion or even a two-nucleotide mutation within the *miR-M4* seed region is sufficient to prevent lymphoma induction (Wang, 2020), while restoration of oncogenicity can be achieved by revertant viruses expressing either MDV-*miR-M4* or the cellular homolog *gga-miRNA*. This landmark finding represented the first direct demonstration that a virus-encoded miRNA is essential for tumor induction in a natural host, firmly establishing miRNA orthologs as bona fide viral oncogenes (Bondada et al., 2019).

**Fig. 2:**
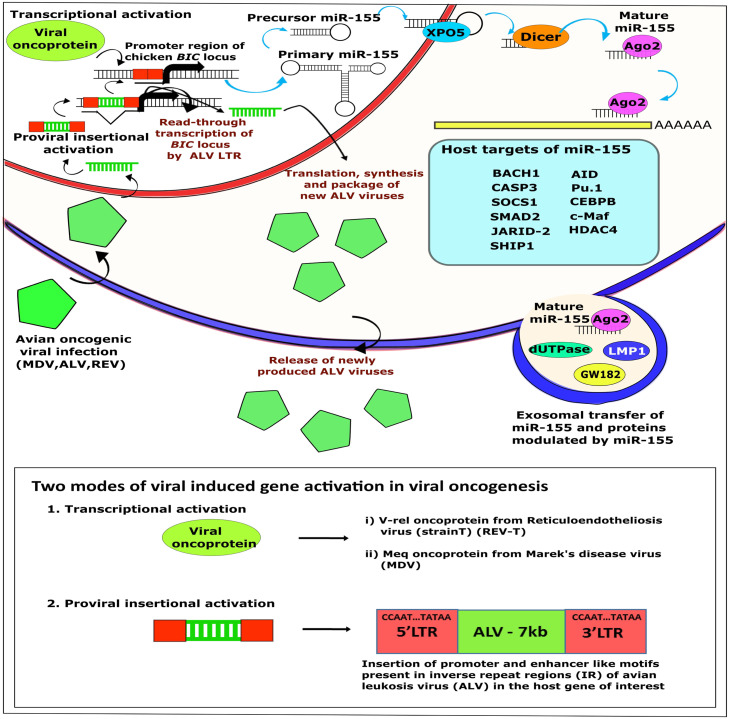
Molecular mechanisms of transformation by oncogenic viruses through miRNA pathways (Bondada et al., 2019).

**Fig. 3:**
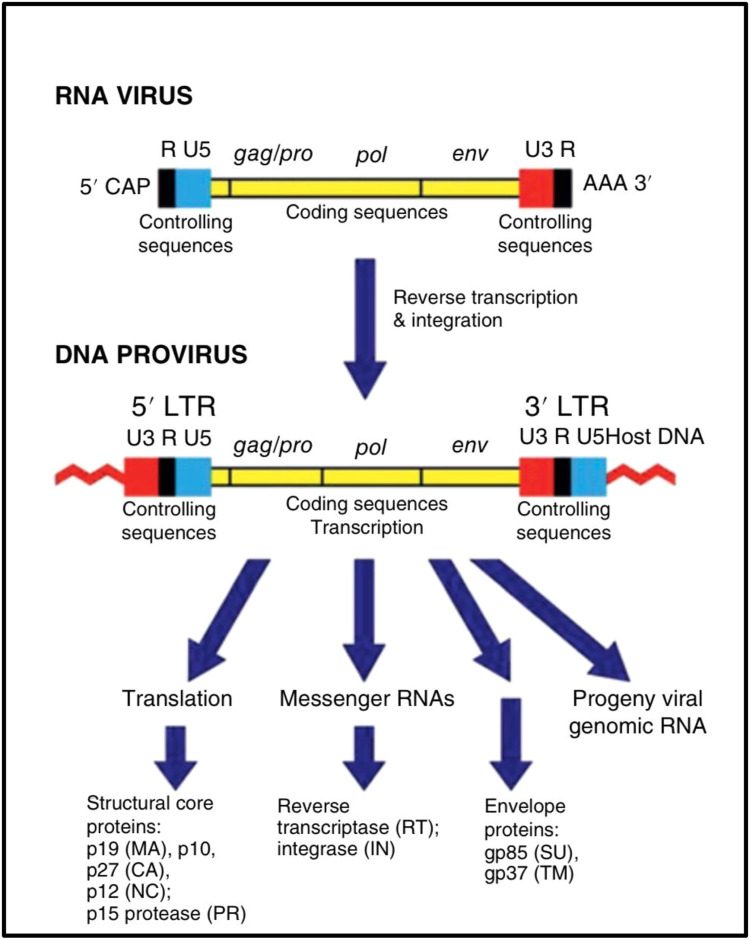
Key features of the viral RNA and proviral DNA forms of the genome of avian leukosis virus (Brugère-Picoux et al., 2015).

The avian leukosis virus, an oncogenic alpharetrovirus of the family Retroviridae, induces a spectrum of leukemia-like neoplastic diseases of the hematopoietic system primarily through insertional mutagenesis (Fotouh et al., 2026). The ALV genome encodes the canonical retroviral structural genes (*gag, pol, and env*) (Fig. 2), and following receptor-mediated entry (Ochi et al., 2012), reverse transcription, and integration of proviral DNA into the host genome, tumorigenesis occurs through activation of cellular proto-oncogenes such as *c-myc, c-erbB, c-myb, and TERT*, resulting in lymphoid, myeloid, or erythroid tumors depending on the target cell population (Duan et al., 2022). Although ALVs are not generally known to encode viral miRNAs, accumulating evidence indicates that they profoundly manipulate the host miRNA landscape to support transformation and tumor maintenance (Gao et al., 2012; Mahmoud et al., 2025). Studies of ALV-transformed cell lines, including DT40 and HD11, revealed widespread dysregulation of host miRNAs, with miRNA, originally identified as the product of the bic gene, frequently upregulated in ALV-induced B-cell lymphomas (Zhou and Zheng, 2019). Given miRNA's established role as a potent oncomiRNA regulating immune responses, inflammation, and myeloid lineage specification, its activation in ALV-induced tumors strongly suggests a contributory role in sustaining the transformed phenotype (Wang et al., 2013). However, unlike MDV-1, where *miR-M4* is indispensable for oncogenesis, the requirement for miRNA in ALV-mediated transformation appears to be context-dependent and not universally essential (Feng et al., 2017). This is illustrated by the observation that certain ALV-derived B-cell lymphoma lines, such as DT-40, do not exhibit elevated miRNA expression, and that CRISPR/Cas9-mediated deletion of miRNA in the ALV-transformed HP45 cell line does not impair continued proliferation, indicating that insertional activation of dominant oncogenes may override miRNA dependency in some settings (Yao et al., 2013). Nevertheless, deep-sequencing approaches have identified a novel small RNA encoded within the X-region small RNA (XSR) element of the ALV-J genome, which is highly expressed in ALV-J–transformed cell lines, raising the possibility that certain ALV strains may encode functional non-coding RNAs that contribute to viral replication or oncogenesis, although their precise biological roles remain to be elucidated (Fotouh et al., 2024). Fig. 3

Reticuloendotheliosis virus, another oncogenic retrovirus of the family Retroviridae, further reinforces the central role of miRNA in avian viral oncogenesis through a distinct but convergent mechanism (Shosha et al., 2024). REV infection is associated with a complex of syndromes in poultry and game birds, including runting disease characterized by growth retardation, bursal and thymic atrophy, immunosuppression, anemia, enteritis, and necrosis of the liver and spleen, as well as chronic lymphoid neoplasms involving the bursa of Fabricius and multiple visceral organs (Zhou et al., 2018). Tumor induction by REV typically occurs through insertional activation of *c-myc,* but a particularly aggressive transforming variant, REV-T, carries the transduced oncogene *v-rel*, a constitutively active member of the *rel/NF-κB* family of transcription factors. *v-Rel*-mediated transformation occurs predominantly through persistent activation of *NF-κB* signaling pathways, leading to profound alterations in host gene expression (Luan et al., 2016).

Notably, *v-Rel* directly induces miRNA expression by binding to *NF-κB-*responsive elements within the bic promoter, resulting in robust upregulation of miRNA in REV-transformed cells (Matos et al., 2022). Transcriptomic analyses of REV-T–transformed chicken B cells revealed extensive enrichment of predicted miRNA target sites among deregulated genes, with downregulation of multiple miRNA target proteins involved in apoptosis, immune regulation, and cell cycle control, thereby supporting a direct functional role for miRNA in neoplastic transformation (Wozniakowski et al., 2015). Interestingly, a striking parallel exists between REV-mediated miRNA induction and Epstein–Barr virus–associated oncogenesis, where the EBV-encoded latent membrane protein-1 also activates miRNA transcription via *NF-κB* signaling, underscoring the convergence of oncogenic viruses on the miRNA regulatory axis (Moore et al., 2000). Consistent with this, upregulation of both *c-myc* and miRNAs has been observed in REV-transformed bursal and splenic tissues, further implicating miRNAs as central mediators linking viral oncogene activity to host transcriptional reprogramming (Bondada et al., 2019). Collectively, studies of MDV-1, ALV, and REV demonstrate that miRNAs and their viral homologs function as conserved molecular hubs that integrate viral oncogenic signals with host pathways governing lymphocyte survival, proliferation, immune evasion, and tumor progression (Wang, 2020; Zavala and Nair, 2020). While the degree of miRNA dependency varies among viruses being essential in MDV-1, contributory but context-dependent in ALV, and strongly inducible through *NF-κB* signaling in REV the recurrent exploitation of the miRNA pathway across these distinct avian oncogenic viruses (Ahsan et al., 2024) underscores its fundamental importance in viral tumorigenesis and highlights miRNA as a unifying mechanistic link between viral infection, immune dysregulation, and cancer development in poultry (Singh et al., 2003).

## The miRNA-mediated oncogenic transformation and immunosuppression

The important aspect that links viral oncogenesis with disease progression in avian oncogenic viruses is the interplay between miRNA-driven cellular transformation and virus-induced immunosuppression (Luan et al., 2016). Although the present review highlights the role of miRNAs in tumor development, accumulating evidence suggests that many of these same miRNAs simultaneously modulate host immune responses, thereby creating a permissive environment for malignant expansion (Wozniakowski et al., 2015). One of the most prominent examples is *miR-155* and its viral ortholog, MDV-miR-M4, which functions as a central regulatory node linking lymphocyte proliferation to immune dysregulation. *miR-155* is widely recognized as a key regulator of immune cell differentiation, cytokine signaling, and inflammatory responses, but sustained overexpression can profoundly alter immune homeostasis. In transformed lymphocytes, *miR-155* promotes proliferation and survival by targeting several negative regulators of signaling pathways (Li et al., 2019).

In the context of Marek’s disease virus infection, the viral miRNA *miR-M4*, which shares the seed sequence with cellular *miR-155*, drives the expansion of transformed CD4⁺ T cells while simultaneously disrupting normal immune regulatory networks (O’Dowd et al., 2020). Downregulation of SHIP1 and SOCS1 by *miR-M4* leads to sustained activation of pathways such as *NF-κB* and JAK/STAT, which not only stimulate lymphocyte proliferation but also impair the regulation of inflammatory responses and immune signaling (Yao et al., 2008). This dysregulation reduces the ability of the host immune system to mount effective antiviral and antitumor responses, allowing transformed lymphocytes to evade immune surveillance and accumulate within peripheral nerves and visceral organs. Consequently, the aggressive lymphoproliferative lesions observed in MDV-1 are not solely a result of uncontrolled cellular proliferation but also reflect a compromised immune microenvironment that fails to eliminate emerging tumor cells (Annese et al., 2020).

A similar interaction between miRNA dysregulation and immune impairment is observed in ALV and REV infections. In ALV-induced tumors, increased expression of *miR-155* has been associated with altered cytokine signaling and impaired differentiation of immune cell populations, contributing to reduced immune responsiveness in infected birds (Ibrahim et al., 2025). REV infection further exemplifies this cross-talk through the activity of the viral oncogene *v-Rel*, which activates NF-κB signaling, thereby inducing *miR-155* transcription via the *bic* promoter (Zlotorynski, 2019). The resulting upregulation of *miR-155* not only supports neoplastic transformation but also interferes with normal immune regulation by suppressing genes involved in apoptosis and immune signaling. These combined effects promote both tumor cell survival and systemic immunosuppression, facilitating viral persistence and disease progression (Glaich et al., 2019).

Therefore, miRNA dysregulation in avian oncogenic viral infections should be viewed not merely as a driver of malignant transformation but as part of a dual pathogenic mechanism in which oncogenic signaling and immune evasion are tightly interconnected (Treiber et al., 2019). By simultaneously enhancing lymphocyte proliferation and weakening host immune surveillance, miRNAs such as *miR-155* and MDV-*miR-M4* create a cellular environment that favors both viral replication and tumor development (Yao et al., 2008). This functional coupling between oncogenic transformation and immunosuppression provides a unifying explanation for the severe lymphoproliferative and immunosuppressive manifestations observed in MDV, ALV, and REV infections, and highlights miRNA regulatory networks as critical determinants of disease outcome (Ibrahim et al., 2025).

## Gross lesions of oncogenic viruses

Gross pathological examination of chickens affected by avian oncogenic viruses reveals a wide spectrum of neoplastic and degenerative lesions that reflect differences in viral tropism, mechanisms of oncogenesis, and host immune responses, yet share several overlapping features characteristic of lymphoid and hematopoietic malignancies (Cui et al., 2013). In infections caused by MDV-1, gross lesions are often dramatic and multisystemic, particularly in birds infected with virulent or very virulent strains (Ibrahim et al., 2025). A lesion of Marek’s disease is the marked enlargement and discoloration of peripheral nerves, especially the sciatic, brachial, vagal, and intercostal nerves, which appear thickened, edematous, and grayish-white, frequently losing their normal striated appearance; these changes correlate clinically with paralysis and paresis (Su et al., 2013). Visceral lymphomas are commonly observed, presenting as diffuse or nodular tumorous infiltrates in organs such as the liver, spleen, kidneys, gonads, heart, lungs, proventriculus, and intestines (Tulman et al., 2000). The liver is often enlarged, with rounded edges, and exhibits multiple gray-white to cream-colored nodules or diffuse pallor due to lymphoid infiltration, while the spleen may be markedly enlarged or, less commonly, atrophic, depending on the disease stage (Zhang et al., 2005). Renal enlargement with distortion of lobulation and compression of adjacent structures is frequently noted, and gonadal tumors may result in irregular enlargement and loss of normal architecture (Parnas et al., 2014). Cutaneous Marek’s disease may manifest grossly as raised nodules associated with feather follicles, producing a roughened skin surface (Morgan et al., 2008). In contrast, the bursa of Fabricius in MD is typically spared or atrophic, aiding in differentiation from other lymphoid neoplasms (Yao et al., 2008).

In ALV-infected birds, gross lesions are more variable and often insidious, reflecting the slower tumor development associated with insertional mutagenesis (Gonda et al., 1981). The most characteristic lesions include lymphoid tumors primarily affecting the bursa of Fabricius in cases of lymphoid leukosis, in which the bursa appears markedly enlarged, firm, and nodular, with a smooth or bosselated external surface (Wang et al., 2020) on cut section, the normal follicular pattern is replaced by uniform gray-white neoplastic tissue (Fotouh et al., 2020). As the disease progresses, metastatic lesions may be observed in the liver, spleen, kidneys, and occasionally the bone marrow, resulting in hepatosplenomegaly with diffuse pallor or discrete tumor nodules (Teng et al., 2017; Abu El Hammed et al., 2022). In erythroid or myeloid leukosis, organs may appear congested and enlarged, and the bone marrow may exhibit pale discoloration due to neoplastic cell replacement (Payne and Nair, 2012). Unlike Marek’s disease, peripheral nerve enlargement is typically absent in ALV infections, and gross lesions tend to be more localized to lymphoid and hematopoietic organs (Gao et al., 2010).

Infections with reticuloendotheliosis virus produce a combination of neoplastic, degenerative, and immunosuppressive lesions, often accompanied by severe growth retardation and poor body condition (Shosha et al., 2024). Grossly, affected birds may exhibit marked runting, emaciation, and poor feather development. Lymphoid tumors induced by REV frequently involve the bursa of Fabricius, spleen, liver, thymus, and other visceral organs, appearing as diffuse enlargement or multifocal gray-white nodules similar to those seen in ALV infections (Mays et al., 2012). The bursa and thymus are often atrophic in immunosuppressive forms of the disease, reflecting profound lymphoid depletion, while in neoplastic forms, these organs may be enlarged and distorted by tumor masses. Hepatomegaly and splenomegaly with mottled or necrotic areas are common, and in some cases, the liver and spleen may exhibit friability due to extensive cellular infiltration and necrosis. Non-bursal T-cell lymphomas may involve multiple organs, including the heart, kidneys, and intestines, leading to organ enlargement and functional compromise. Collectively, MDV-1, ALV, and REV share common gross features, such as visceral lymphoid tumors and organ enlargement, but careful evaluation of nerve involvement, bursal changes, lesion distribution, and overall body condition provides critical clues for differential diagnosis during post-mortem examination of oncogenic viral diseases in chickens.

## Microscopic lesions of oncogenic viruses

Histopathological examination of tissues from chickens affected by avian oncogenic viruses reveals distinctive yet partially overlapping microscopic lesions that reflect differences in viral tropism, mechanisms of cellular transformation, and patterns of immune modulation (Ibrahim et al., 2025). In MDV-1 infections, microscopic lesions are characterized by pleomorphic lymphoid infiltrates predominantly composed of transformed T lymphocytes at various stages of differentiation (Xu et al., 2011). A feature is the presence of dense, diffuse, or multifocal infiltrates of pleomorphic lymphoid cells within peripheral nerves, particularly the sciatic and brachial nerves (Cui et al., 2010), resulting in marked loss of normal nerve architecture, demyelination, axonal degeneration, and replacement of nerve fibers by neoplastic lymphocytes and macrophages (Coupeau et al., 2012). In visceral organs such as the liver, spleen, kidneys, heart, lungs, gonads, and proventriculus, MDV-1 induces diffuse or nodular lymphomas composed of medium to large lymphoid cells with hyperchromatic nuclei, prominent nucleoli, high mitotic activity, and frequent atypical mitoses (Davidson et al., 2002). The infiltrates often extend beyond normal tissue boundaries, causing compression and atrophy of surrounding parenchyma (Hicks and Liu, 2013). In the skin, lymphoid cells infiltrate feather follicles and dermis, sometimes accompanied by epidermal hyperplasia (Schat, 2004). Notably, the bursa of Fabricius in Marek’s disease is typically depleted or atrophic rather than neoplastic, a key histological feature useful for differential diagnosis (Yao et al., 2007).

Avian leukosis virus induces tumors primarily through insertional mutagenesis (Zhou et al., 2019), and its microscopic lesions are generally more uniform and monomorphic compared to those of MDV-1. In lymphoid leukosis, the bursa of Fabricius is the principal target organ, where normal follicular architecture is effaced by sheets of uniform, well-differentiated lymphoblastoid cells of B-cell origin (Boumart et al., 2018). These cells typically display round nuclei with finely stippled chromatin, inconspicuous nucleoli, and moderate basophilic cytoplasm, with relatively lower pleomorphism than MDV-1 associated lymphomas (Zhao et al., 2022). As the disease progresses, neoplastic cells infiltrate the liver, spleen, kidneys, and bone marrow, leading to diffuse replacement of normal hematopoietic and parenchymal tissues (Stelekati et al., 2018). In myeloid or erythroid leukosis, bone marrow hypercellularity is prominent, with excessive proliferation of immature hematopoietic precursors, sinusoidal congestion, and displacement of normal cell lineages (Fotouh et al., 2024). Peripheral nerve involvement is absent in ALV infections, and inflammatory reactions are generally minimal, reflecting the slower, less inflammatory nature of ALV-induced tumor development (Cai et al., 2009).

Reticuloendotheliosis virus produces a spectrum of microscopic lesions that combine features of neoplasia, immunosuppression, and chronic inflammation (Yu et al., 2007). In immunosuppressive forms, severe lymphoid depletion is evident in primary lymphoid organs, including the bursa of Fabricius and the thymus, where marked cortical thinning, follicular atrophy, and lymphocyte depletion are observed, often accompanied by increased numbers of macrophages and reticular cells (Yao et al., 2017). In neoplastic forms of REV infection, particularly those associated with the acutely transforming REV-T strain, tissues exhibit diffuse or nodular infiltrates of neoplastic lymphoid or reticuloendothelial cells, frequently involving the bursa, spleen, liver, thymus, heart, and kidneys (Bolisetty et al., 2009). These neoplastic cells may display variable morphology, ranging from relatively uniform lymphoblasts to pleomorphic populations depending on the viral strain and target cell lineage, and often show high mitotic indices (Niewiadomska and Gifford, 2013). Histological sections frequently reveal extensive disruption of normal tissue architecture, vascular invasion by tumor cells, and areas of necrosis (Kim et al., 2011). In REV-induced tumors, activation of NF-κB–associated pathways correlates with increased cellular proliferation and resistance to apoptosis, features that are reflected histologically by dense cellularity and limited apoptotic bodies (Sun et al., 2007). While MDV-1, ALV, and REV share the common histopathological theme of lymphoid or hematopoietic neoplasia, careful assessment of cellular morphology, degree of pleomorphism, involvement of peripheral nerves, status of the bursa of Fabricius, and presence or absence of lymphoid depletion provides critical diagnostic criteria for distinguishing among these oncogenic viral diseases during microscopic evaluation (Van et al., 2012).

## Discussion and conclusion

The collective evidence reviewed in this work underscores the central role of microRNA and its viral homologs as key molecular regulators in the pathogenesis and pathology of avian oncogenic viral diseases, particularly those caused by Marek’s disease virus, avian leukosis virus, and reticuloendotheliosis virus (Bondada et al., 2019). Although these viruses differ markedly in genomic organization, replication strategies, and mechanisms of cellular transformation, they converge on conserved host regulatory pathways that govern lymphocyte proliferation, survival, immune modulation, and tumor development (Ibrahim et al., 2025). Among these pathways, microRNA emerges as a unifying oncogenic hub linking viral infection to pathological outcomes at both the cellular and tissue levels (Jones-Rhoades and Bartel, 2004).

One of the most striking observations arising from studies on MDV-1 is that viral oncogenesis can be driven directly by a virus-encoded miRNA rather than by a classical protein oncogene (Ibrahim et al., 2025). MDV-1–encoded *miR-M4*, a functional ortholog of cellular microRNA, represents the first experimentally validated example of a viral miRNA that is essential for tumor induction in a natural host (Lagos-Quintana et al., 2001). The complete loss of oncogenicity following deletion or seed-region mutation of *miR-M4* provides compelling in vivo evidence that miRNA-mediated post-transcriptional regulation is not merely a supportive mechanism but can function as a primary oncogenic driver (Quarton et al., 2018). This finding has profound implications for avian tumor pathology, as it helps explain the aggressive lymphoproliferative lesions observed in Marek’s disease, including pleomorphic T-cell lymphomas, extensive visceral infiltration, and severe peripheral nerve involvement (Yao et al., 2008). Histopathologically, the high mitotic index, marked cellular pleomorphism, and widespread tissue invasion characteristic of MDV-1 associated lymphomas are consistent with sustained miRNA-like activity, which promotes cell survival, inhibits apoptosis, and enhances proliferative signaling (Wang, 2020).

In contrast, ALV-induced oncogenesis follows a slower, more insidious pathological course, primarily driven by insertional mutagenesis and the activation of host proto-oncogenes such as c-myc, c-erbB, and TERT (Zhou and Zheng, 2019). Nevertheless, the frequent upregulation of miRNA in ALV-induced B-cell lymphomas suggests that this oncomiRNA plays an important modulatory role in maintaining the transformed phenotype (Wang et al., 2013). Unlike MDV-1, in which miRNA ortholog activity is indispensable for tumor initiation, ALV appears to use miRNAs as secondary amplifiers of oncogenic signaling (Fotouh et al., 2026). This distinction is reflected pathologically by the relatively monomorphic nature of ALV-associated lymphomas, particularly in the bursa of Fabricius, and the absence of peripheral nerve lesions (Fotouh et al., 2020). The observation that miRNA deletion does not universally abrogate the transformed phenotype in ALV-derived cell lines underscores the complexity of retrovirus-induced tumorigenesis, in which miRNA dysregulation acts in concert with dominant insertional oncogenic events rather than serving as the sole driver (Ibrahim et al., 2025).

REV-induced disease further reinforces the pathological significance of miRNA, particularly in the context of immune dysregulation and NF-κB–mediated transformation (Sun et al., 2007). The acutely transforming REV-T strain, which carries the viral oncogene v-rel, exemplifies how viral activation of host transcriptional programs can directly induce miRNA expression through *NF-κB* binding sites in the bic promoter (Moore et al., 2000). This mechanism establishes a direct link between viral oncogene expression, miRNA upregulation, and pathological outcomes. Microscopically, REV-associated lesions often combine features of neoplasia and immunosuppression, including lymphoid depletion, reticuloendothelial hyperplasia, and diffuse lymphoid infiltrates affecting multiple organs (Shosha et al., 2024). The enrichment of miRNA target genes among deregulated transcripts in REV-transformed cells supports a model in which microRNA acts as a downstream effector of *NF-κB* signaling, promoting tumor cell survival and resistance to apoptosis while simultaneously impairing normal immune function (Cui et al., 2010).

From a comparative pathology perspective, the differences in gross and microscopic lesions observed among MDV-1, ALV, and REV can be interpreted through the lens of miRNA dependency and regulatory dominance (Faiz et al., 2017). MDV-1–associated tumors exhibit marked pleomorphism, aggressive tissue invasion, and peripheral nerve involvement, consistent with strong and sustained microRNA-like activity mediated by *miR-M4* (Zhou and Zheng, 2019). ALV-associated tumors, by contrast, are typically more localized, monomorphic, and bursa-centered, reflecting a primary reliance on insertional oncogene activation, with variable contributions from miRNAs (Duan et al., 2022). REV-associated lesions occupy an intermediate pathological spectrum, characterized by both neoplastic transformation and profound lymphoid depletion, aligning with *NF-κB*–driven miRNA induction and immune suppression (Shosha et al., 2024). These distinctions highlight the value of integrating molecular findings with pathological observations to improve differential diagnosis and disease classification in avian oncology (Gao et al., 2010).

Importantly, the conservation of miRNA–mediated regulatory networks across species underscores the broader relevance of avian oncogenic viruses as models for human virus-associated cancers (Zhou and Zheng, 2019). The shared targeting of genes such as JARID2, SOCS1, SHIP1, and NF-κB-related signaling components by cellular microRNA and viral orthologs from MDV-1, Epstein–Barr virus, and Kaposi’s sarcoma–associated herpesvirus illustrates an evolutionarily conserved strategy by which oncogenic viruses exploit host miRNA pathways to drive malignant transformation (Cai et al., 2009). This evolutionary conservation not only validates the biological significance of microRNA in tumor pathology but also positions avian models as valuable systems for studying fundamental mechanisms of viral oncogenesis (Bondada et al., 2019).

From a diagnostic and translational standpoint, the prominent role of microRNAs in avian tumor pathology suggests their use as molecular biomarkers. Differential expression of miRNAs or their viral homologs may aid in distinguishing MDV, ALV, and REV associated tumors, particularly when gross or histological features overlap (Bondada et al., 2019). Moreover, miRNA expression patterns could provide insights into tumor aggressiveness, immune status, and disease progression (Hicks and Liu, 2013). Therapeutically, although direct targeting of miRNAs in poultry is currently impractical, understanding miRNA-driven pathways may inform vaccine design, genetic resistance strategies, and improved disease control measures aimed at limiting viral persistence and oncogenic evolution (Jones-Rhoades and Bartel, 2004).

Beyond their diagnostic and pathogenic significance, miRNAs have increasingly been explored as potential targets for novel control strategies against avian oncogenic viruses (Zhou and Zheng, 2019). Advances in molecular virology and genome editing technologies have opened several promising avenues that leverage miRNA biology to limit viral replication, oncogenesis, and immunosuppression in poultry (Yakovlev, 2019). One emerging strategy involves the use of miRNA inhibitors, such as antisense oligonucleotides or antagomirs, designed to neutralize oncogenic miRNAs (Li et al., 2020). Experimental studies targeting *miR-155* or its viral ortholog MDV-*miR-M4* have demonstrated that suppression of these miRNAs can significantly reduce proliferation of transformed lymphoid cells and restore the expression of tumor-suppressive targets (Ma et al., 2020). Although the application of miRNA inhibitors in poultry remains largely experimental, such approaches have shown promise in mammalian cancer models and may represent a potential therapeutic avenue for controlling virus-induced lymphomas in high-value breeding flocks or experimental systems (Wu et al., 2019). A second approach involves engineering viral vaccines to exploit host miRNA expression patterns. In this strategy, viral genomes are modified to include target sequences for host-specific miRNAs, causing viral transcripts to be selectively degraded in certain tissues or immune cell populations (Ibrahim et al., 2025). This technique has been successfully used in several viral vaccine platforms to attenuate viral replication while maintaining immunogenicity. In the context of avian oncogenic viruses, incorporating chicken-specific miRNA target sites into recombinant vaccine strains could potentially improve vaccine safety by limiting replication in lymphoid tissues where oncogenic transformation occurs (Chen et al., 2020). A third and increasingly discussed strategy is the development of genetically modified or selectively bred chickens with altered miRNA expression profiles that confer resistance to viral oncogenesis (Zhou and Zheng, 2019). Experimental models have suggested that modulation of miRNAs involved in immune regulation, including miR-155 and other immune-associated miRNAs, may enhance antiviral responses and improve resistance to tumor development following MDV infection (Fotouh et al., 2026). In addition to genome editing, selective breeding programs based on genomic markers linked to miRNA-regulated immune pathways may provide a practical approach to improving disease resistance in commercial poultry populations. Together, these emerging strategies illustrate how insights into miRNA-mediated regulatory networks may translate into innovative approaches for controlling avian oncogenic viral diseases (Gao et al., 2010).

In conclusion, the reviewed literature collectively demonstrates that miRNAs and their viral homologs occupy a central role in the pathogenesis and pathology of avian oncogenic viral diseases. By integrating molecular regulation with gross and microscopic pathological findings, this discussion highlights miRNA as a critical link between viral infection, immune dysregulation, and tumor development. Future research should focus on dissecting tissue-specific microRNA functions, host genetic modifiers of miRNA activity, and the interplay between viral evolution and miRNA-mediated oncogenic pathways, thereby advancing both our understanding of avian tumor pathology and the broader field of virus-associated cancer biology. Another major gap concerns the tissue-specific functions of viral microRNAs, particularly MDV-*miR-M4*, which is known to be essential for tumor induction but whose regulatory roles may vary across infected tissues. For example, the mechanisms by which MDV-miR-M4 contributes to lymphocyte transformation in visceral organs compared with its potential role in peripheral nerve lesions, one of the hallmark pathological features of Marek’s disease, remain poorly understood. Clarifying whether distinct miRNA target networks operate in neural versus lymphoid tissues may provide important insights into the pathogenesis and tissue tropism of MDV-induced tumors.

Another unresolved question relates to the functional significance of newly identified small RNAs associated with avian leukosis virus, particularly those encoded within the X region of ALV-J genomes. In addition, further research is needed to elucidate the interactions between viral miRNAs and host immune regulatory pathways, particularly the mechanisms by which miRNA-mediated signaling simultaneously promotes lymphocyte proliferation and suppresses antiviral immune responses.

## Funding

No funding

## CRediT authorship contribution statement

**Ahmed Fotouh:** Writing – original draft, Supervision, Methodology. **Rania M. Elbatawy:** Writing – review & editing, Resources. **Ibrahim Eldaghayes:** Writing – review & editing, Investigation, Funding acquisition. **Eman Abd El‑Menamm Shosha:** Writing – review & editing, Methodology.

## Disclosures

All authors declare that there is no conflict of interest
